# Compromised Cortical-Hippocampal Network Function From Transient Hypertension: Linking Mid-Life Hypertension to Late Life Dementia Risk

**DOI:** 10.3389/fnins.2022.897206

**Published:** 2022-06-23

**Authors:** Aaron Y. Lai, Paolo Bazzigaluppi, Christopher D. Morrone, Mary E. Hill, Bojana Stefanovic, JoAnne McLaurin

**Affiliations:** ^1^Biological Sciences, Sunnybrook Research Institute, Toronto, ON, Canada; ^2^Department of Medical Biophysics, University of Toronto, Toronto, ON, Canada; ^3^Physical Sciences, Sunnybrook Research Institute, Toronto, ON, Canada; ^4^Department of Laboratory Medicine and Pathobiology, University of Toronto, Toronto, ON, Canada

**Keywords:** hypertension, F344 rats, myelin, prefrontal cortex, hippocampus, network coherence, cognitive flexibility

## Abstract

Mid-life hypertension is a major risk factor for developing dementia later in life. While anti-hypertensive drugs restore normotension, dementia risk remains above baseline suggesting that brain damage sustained during transient hypertension is irreversible. The current study characterized a rat model of transient hypertension with an extended period of normotensive recovery: F344 rats were treated with L-NG-Nitroarginine methyl ester (L-NAME) for 1 month to induce hypertension then allowed up to 4 months of recovery. With respect to cognitive deficits, comparison between 1 month and 4 months of recovery identified initial deficits in spatial memory that resolved by 4 months post-hypertension; contrastingly, loss of cognitive flexibility did not. The specific cells and brain regions underlying these cognitive deficits were investigated. Irreversible structural damage to the brain was observed in both the prefrontal cortex and the hippocampus, with decreased blood vessel density, myelin and neuronal loss. We then measured theta-gamma phase amplitude coupling as a readout for network function, a potential link between the observed cognitive and pathological deficits. Four months after hypertension, we detected decreased theta-gamma phase amplitude coupling within each brain region and a concurrent increase in baseline connectivity between the two regions reflecting an attempt to maintain function that may account for the improvement in spatial memory. Our results demonstrate that connectivity between prefrontal cortex and hippocampus is a vulnerable network affected by transient hypertension which is not rescued over time; thus demonstrating for the first time a mechanistic link between the long-term effects of transient hypertension and dementia risk.

## Introduction

Dementia affects more than 57 million people worldwide in 2019, a number expected to triple by 2050 (GBD 2019 Dementia Forecasting Collaborators, 2022). Hypertension is increasingly recognized as an important risk factor for cognitive decline and dementia (Walker et al., 2017). The link between hypertension and a wide array of cognitive deficits is well documented (Gorelick et al., 2011); however, the pathophysiological mechanism underlying this link remains unresolved. In aging studies, people who developed dementia had a history of hypertension (Skoog et al., 1996; Tzourio et al., 2014). A prospective study found that treatment of mid-life hypertension dampened the decline in memory, but failed to restore it to levels of healthy aging controls (Köhler et al., 2014). This suggests that transient hypertension induces permanent injury to neurons and neuronal networks. Clinical cases of hypertension are mostly transient in nature as patients receive anti-hypertensive treatment (Joffres et al., 2013). There is evidence suggesting that while clinical hypertension is not persistent, it is sufficient to induce irreversible brain damage that contributes to later cognitive impairment: several studies have found that anti-hypertensive treatment does not return risk of dementia to baseline levels (in’t Veld et al., 2001; Khachaturian et al., 2006; Yasar et al., 2013; Walker et al., 2017; Xu et al., 2017). Important questions remain with regard to the specific brain regions, neuronal circuits, and cellular processes affected by transient hypertension.

Animal models of chronic hypertension are well established and have been used to examine brain impairment; these models include genetically hypertensive rat strains and constriction of blood vessels surgically or pharmacologically (Díaz-Ruiz et al., 2009; Cifuentes et al., 2015, 2017; Kruyer et al., 2015; Shih et al., 2018; Levit et al., 2020). Nonetheless, the majority of experimental paradigms induce hypertension throughout the experimental period, and the effects of a transient episode of hypertension are rarely examined. Here, we model transient hypertension by treating F344 rats with *N-*nitro-L-arginine methyl ester (L-NAME) (Leong et al., 2015; Bazzigaluppi et al., 2019; Lai et al., 2021) for 1 month followed by 4 months of normotensive recovery. L-NAME induces hypertension through modulation of nitric oxide signaling and vascular tone (Lerman et al., 2019) without long-term impairment of NOS activity (Pechánová et al., 2004). The extended recovery time allowed us to differentiate the specific neuronal circuits and cognitive processes that are most vulnerable to sustained damage from transient hypertension from those that are only transiently affected. Specifically, the prefrontal cortex (PFC) and hippocampus (HIPP) form a critical circuit necessary for both short- and long-term memory; its dysfunction leads to dementia (Sigurdsson and Duvarci, 2016; Sampath et al., 2017). We conducted a combination of behavioral, pathological and electrophysiological assays to examine the effects of transient hypertension on this neuronal network.

## Materials and Methods

### Animals

Age-matched F344 rats were kept on a 12 hour:12 hour light/dark cycle with water and food *ad libitum*. Each cage houses two cage-mates and receives daily monitoring for health and normal grooming behaviors. Ethical approval of all experimental procedures was granted by The Animal Care Committee of the Sunnybrook Health Sciences Center, which adheres to the Policies and Guidelines of the Canadian Council on Animal Care, the Animals for Research Act of the Provincial Statute of Ontario, and the Federal Health of Animals Act.

### Induction of Transient Hypertension

Four-month-old rats were treated with 10 mg/kg/day (males weighing 0.38–0.43 kg) and 7.5 mg/kg/day (females weighing 0.23–0.28 kg) L-NAME in drinking water for 1 month, respectively. These doses were established previously (Lai et al., 2021) such that hypertension is induced without visible effects on normal cage and grooming behavior. Drug exposure was estimated by measuring water consumption in each cage. Hypertension was confirmed by measuring systolic blood pressure at 5, 6, and 9 months of age using the CODA-HT2 tail-cuff system under isoflurane anesthesia (Kent Scientific). Statistical significance was measured by *t*-test.

### Barnes Maze

We conducted all trials except the training of Barnes maze (Maze Engineers) testing in a behavioral suite with spatial cues and an aversive light. Following training to the location of the escape, rats learned the task across 3 days with two trials per day. We assessed spatial memory 3 days later in one probe trial. The next day reversal learning (5 days, two trials per day) for assessing executive function began, in which the location of the escape hole was switched without re-training. Latency to find the escape hole was measured, and search strategies were manually scored by an investigator blinded to treatment conditions as outlined previously (Illouz et al., 2016; Morrone et al., 2020): Direct (1), corrected (0.75), long correction (0.5), focused search (0.5), serial (0.25), and random (0). EthoVision XT (Noldus, version 11.5) was used to collect and analyze video data. Representative heatmaps for these six search strategies are shown in Supplementary Figure 1. GraphPad Prism 6 was used to compute statistical analyses. For data with multiple time points, statistical significance was measured using repeated measures ANOVA with correction for multiple comparisons followed by Holm-Sidak *post hoc*. For search strategy complexity rank, Mann–Whitney *U* test was used. All other data sets were measured using *t*-test. Sex ratios for each experimental group were: Untreated (NTx) 1 month-recovery = 8 females and 8 males; L-NAME 1 month-recovery = 8 females and 5 males; NTx 4 months-recovery = 5 females and 7 males; L-NAME 4 months-recovery = 4 females and 6 males. Two-way ANOVA indicated that sex was not a contributing factor in either the probe or reversal trial readouts for both cohorts: Latency/probe/1 month-recovery (*p* = 0.979); Time in Old Quadrant/reversal/1 month-recovery (*p* = 0.821); Time in Old Quadrant/reversal/4 months-recovery (*p* = 0.968). All experiments in this study thus used sex-balanced cohorts.

### Immunofluorescence, Myelin Staining, and Immunoblotting

#### Immunofluorescence and Myelin Staining

At the end of the experimental period (9 months of age), rats (NTx = 4 females and 4 males; L-NAME = 3 females and 5 males) were transcardially perfused with phosphate-buffered saline (PBS)-0.1% heparin followed by PBS-4% paraformaldehyde. Brains were extracted, post-fixed overnight in PBS-4% paraformaldehyde, cryopreserved in PBS-30% sucrose, then sectioned coronally at 40 μm of thickness between AP + 4.0 to −6.0 mm. Three evenly spaced coronal sections from each of the prefrontal cortex (AP + 4.0 to + 2.5 mm) and hippocampus (AP −2.5 to −4.5 mm) were used for labeling and quantification.

For fluorescent labeling, floating sections were blocked for 1 h, probed with primary antibodies overnight at 4°C, then incubated with both fluorescence-tagged secondary antibodies and fluorescence-tagged tomato lectin (Vector #TL-1176-1, 1:250) for 2 h. Both the block and antibody incubations were diluted in PBS-0.5% Triton-0.5% bovine serum albumin. The following antibodies were used: Anti-aquaporin-4 (Millipore #AB3594, 1:500), anti-myelin basic protein (Millipore #MAB386, 1:50), anti-NeuN (Millipore #ABN90, 1:250), anti-rabbit IgG Alexa488 (Life Technologies #A21206, 1:250), anti-rat IgG Alexa594 (Life Technologies #A11007, 1:250), and anti-guinea pig IgG Alexa594 (Jackson #706-585-148, 1:250).

For staining of myelin fibers we used Black Gold II (Histo-Chem #1BGII). Sections were first mounted on slides, then incubated with 1 mL of 0.3% Black Gold II (in saline) on a heating block at 60 °C for 5 min. The slides were then washed and incubated with 1 mL of 1% sodium thiosulfate (in saline) for 3 min before alcohol dehydration and coverslipping.

Whole brain images of labeled sections were acquired with the Zeiss Observer.Z1. Investigator acquiring the images was blinded to the rat numbers. ImageJ was used to quantify density of fluorescence labeling and myelin staining. Each image was blinded to the investigator performing the quantification: First, regions of interest were drawn to include dorsal PFC and whole HIPP (Supplementary Figure 2). Total pixels in the drawn regions were summed using “Analyze Particles” after thresholding background intensity to 5–8 pixels per 500 μm^2^. Area coverage/density for each stain/marker (percentage area covered by pixels) was then expressed as % area. To count the number of NeuN-positive cells, objects > 25 pixels^2^ were summed using “Analyze particles” after auto-thresholding (Default algorithm) within each region of interest. To count the number of vessels, objects > 200 pixels^2^ were summed using “Analyze Particles” after thresholding background intensity to 10–15 pixels per 100 μm^2^ within each region of interest. These parameters successfully counted > 95% of all vessels > 5 μm in diameter. GraphPad Prism 6 was used to compute statistical significance which was measured using *t*-test.

#### Immunoblotting

Protein extraction from paraformaldehyde-fixed tissue sections were performed according to a previously established protocol (Thacker et al., 2021). In brief, eight sections from the same rats used for immunofluorescence containing either PFC or HIPP were pooled and sonicated for three times (five seconds each) in lysis buffer [100 mM NaCl, 25 mM EDTA, 500 mM Tris–HCl, 1% Triton, 1% NP-40, 2% SDS, and 1% protease inhibitor (Millipore #539134)]. Sonicated lysates were agitated at 90°C for 2 h then centrifuged at 1,000 × *g*. Supernatants were subjected to SDS-PAGE electrophoresis and immunoblotting which have been previously described in detail (Joo et al., 2017). Anti-aquaporin-4 (Millipore #AB3594, 1:1,000) and anti-GAPDH (Sigma #G9545, 1:100,000) were used. Densitometry analysis of immunoblots was carried out by an experimenter blinded to treatment conditions using ImageJ; density values were obtained by multiplying Mean Gray Value (minus background) and Area. GraphPad Prism 6 was used to compute statistical significance which was measured using *t*-test.

### Electrophysiology

#### Recordings

Animals (NTx = 3 females and 2 males; L-NAME = 2 females and 3 males) were anesthetized with isofluorane (5% induction and 1–1.5% maintenance) and positioned in a stereotaxic frame (David KOPF Instruments). An intravenous catheter was inserted in the tail vein for propofol delivery during the recordings (bolus 7.5 mg/kg followed by continuous intravenous infusion 44.0 mg/kg/h). Two cranial windows were created in the same hemisphere, centered over the prefrontal cortex (AP + 3.5 mm, ML ± 0.5 mm) and somatosensory region (AP-3.0 mm, ML ± 0.5 mm). At the end of the surgery, isoflurane was gradually discontinued and animals were administered low dose of propofol to maintain a state of light sedation. A two-shank linear multielectrode array was lowered into each window. Each shank was equipped with one platinum/iridium recording site (200 μm in diameter, Microprobes for LifeScience). Resting-state local field potentials were amplified (Model 3600, AM-systems) between 0.3 Hz and 5 kHz, sampled at 20 kHz (DataWave) and stored on a desktop computer for offline analysis.

#### Data Analysis

Power line noise (60 Hz) and its harmonics were removed *via* notch filtering before additional analysis using a zero-phase forward and reverse Butterworth infinite impulse response filter with frequency range of ± 2 Hz around the stop band (filtfilt.m in Matlab; MathWorks) to eliminate phase distortions. Recordings were re-referenced offline between the two recording sites within every brain area. For estimation of Phase Amplitude Coupling, we followed the original formulation of Tort et al. (2010). The frequency band of interest were theta (3–9 Hz), alpha (10–14 Hz), beta (15–30 Hz), low gamma (30–58) and high gamma (62–120 Hz). These frequency bands have been hypothesized to arise from dynamic motifs that are based on structural circuits and that dictate behavioral function, after input-output transformation (Womelsdorf et al., 2014). The phenomenon by which one frequency band can modulate the activity of a different frequency band has been observed in both animal and human data and is termed cross-frequency coupling (Canolty and Knight, 2010). The events of interest in cross-frequency coupling are features of the ongoing oscillatory activity itself; therefore, cross-frequency coupling refers to statistical dependence between distinct frequencies bands of the ongoing electrical activity rather than dependence of the electrical activity on external stimulus events. Specifically, the theta rhythm can modulate the gamma power of the intracortical local field potential (Bragin et al., 1995; Chrobak and Buzsáki, 1998; Lee et al., 2005). High-gamma activity is modulated by sensory, motor, and cognitive events (Crone et al., 1998), is functionally distinct from low gamma, and has distinct physiological origins (Edwards et al., 2005). Here, we focus on the phase-amplitude coupling and use an index of cross-frequency coupling referred to as modulation index (MI) that combines the amplitude envelope of time series (A) at different time lag (τ): A1 (t + τ) of a high-frequency band with the phase time series φ2(t) of a low-frequency band into one composite, complex-valued signal z (t, τ). The temporal mean of this composite signal provides a sensitive measure of the coupling strength and preferred phase between the two frequencies (Canolty et al., 2006). The MI was estimated with Matlab Toolbox^1^ (Onslow et al., 2011).

To estimate cross-frequency coupling, we adopted the method presented by Canolty et al. (2006), a population of 50 shuffled signals were created and compared to the original signal to generate a distribution of MI values. MI values lying in the top 5% of this distribution (after Bonferroni correction) were deemed significant. Theta band was divided into 0.5 Hz bins, while both gamma bands were binned every 2 Hz.

The coherence between the EEG channel in the hippocampus and the one in medial prefrontal cortex was measured by amplitude squared coherence (mscohere.m function in Matlab signal toolbox), which computes coherence of the input signals x and y using Welch’s averaged, modified periodogram method. The magnitude squared coherence estimate Cxy(f) indicates how well x corresponds to y at each frequency:

urn:x-wiley:00223042:media:jnc14136:jnc14136-math-0001

where Pxx(f) and Pyy(f) are the power spectral densities of the input signals x and y and Pxy is the cross-power spectral density. For each subject, the mean coherence between the electrode in the hippocampus and in the medial prefrontal cortex was calculated. Specifically, the local field potential signal was segmented into 3-s long, non-overlapping, epochs and the magnitude squared coherence estimated for each epoch and frequency bin for each animal. The bins were 0.3 Hz wide and spanned the frequency range from 2 to 120 Hz.

#### Statistical Analysis

Statistical analysis was performed in R environment (v 4.0.5^2^). We used linear mixed effects modeling (lme function) to model power and coherence as linear functions of treatment (non-treated vs. L-NAME) and, in case of spontaneous activity power and coherence, frequency band, with subjects treated as random effects, thus accounting for across-subject variation. Analysis of variance was performed to assess the interaction between genotype and frequency band.

## Results

**FIGURE 1:**
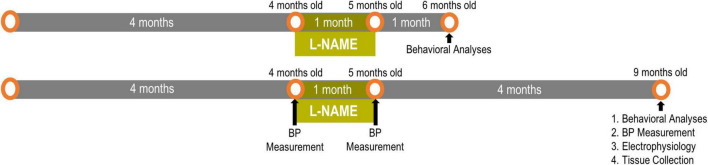
Experimental timeline for L-NAME-induced transient hypertension: One cohort had 1 month of normotensive recovery and the other cohort had 4 months of recovery. Readouts were sequential with behavioral analyses preceding blood pressure and electrophysiological measurements.

We established transient hypertension by treating adult F344 rats with L-NAME (Bazzigaluppi et al., 2019; Lai et al., 2021) between 4 and 5 months of age then ceased treatment at 5 months of age to restore blood pressure to normotension followed by aging to either 6 or 9 months of age (Figure 1). Previous studies have shown that L-NAME effects are resolved within 24 h of cessation of treatment (Salter et al., 1995). To accurately model transiently hypertensive patients (Cigolini et al., 1995; Jeng, 2003), blood pressure was monitored according to our previous studies on F344 rats (Bazzigaluppi et al., 2019; Lai et al., 2021). Blood pressure readings were taken pre-treatment (4 months of age), immediately post-treatment (5 months of age) and after long term recovery (9 months of age after completion of behavioral experiments) (Figure 1). One month of L-NAME treatment elevated blood pressure from 114 ± 4/80 ± 5 mmHg to 151 ± 5/108 ± 5 mmHg in F344 rats (*p* = 0.005), while after cessation of treatment and the recovery period blood pressure was normalized in L-NAME treated rats to 114 ± 5/75 ± 5 mmHg (*p* = 0.99). Untreated rats (NTx) underwent blood pressure measurements and showed no change in their blood pressure over time (4 months 113 ± 6/79 ± 6; 5 months 111 ± 7/81 ± 4; and 9 months of age 103 ± 3/72 ± 1; *p* = 0.21). These results demonstrate the physiological relevance and the transient nature of the hypertensive event in F344 rats in this paradigm.

### Transient Hypertension Induced Loss of Cognitive Flexibility

**FIGURE 2:**
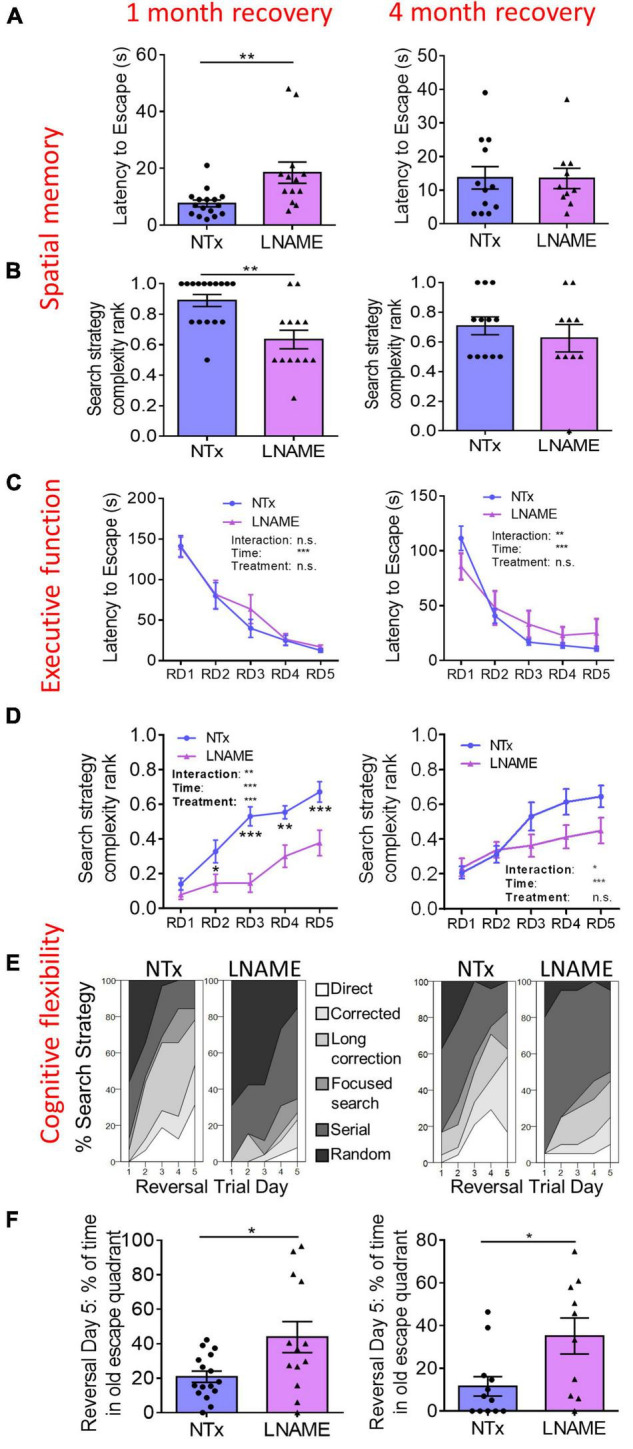
Cognitive functions are impaired in rats 1 month after transient hypertension and are partially restored after 4 months. **(A,B)** In the probe trials, hypertension increased latency to escape (*p* = 0.006) and decreased search strategy complexity (*p* = 0.003) 1 month after normotensive recovery (left) demonstrating deficits in spatial memory. Four months after normotensive recovery (right), these deficits were no longer present (*p* = 0.971 for latency to escape, *p* = 0.631 for search strategy complexity). **(C)** In the reversal trials, hypertension did not affect executive function at both 1 month (*p* = 0.631) and 4 months (*p* = 0.692) recovery as two way repeated measures ANOVA did not show a treatment effect. **(D)** In the reversal trials, hypertension decreased search strategy complexity (*p* = 0.008, two way ANOVA treatment effect) at 1 month recovery but did not at 4 months recovery (*p* = 0.132, two way ANOVA treatment effect) suggesting that cognitive flexibility was initially impaired by hypertension yet recovered after 4 months. **(E)** In the reversal trials, percent distribution of various levels of search strategy complexity showed that at 4 months recovery, although L-NAME rats noticeably improved their search strategies compared to L-NAME rats at 1 month recovery, their search strategies were still less complex than NTx rats suggesting that cognitive flexibility remains partially impaired. **(F)** In the reversal trials, L-NAME rats spent significantly more time in the old escape quadrant at both 1 month (*p* = 0.004) and 4 months (*p* = 0.018) recovery suggesting that deficits in cognitive flexibility did not recover. *, **, and *** denote *p* < 0.05, 0.01, 0.001 respectively; *n* = 16 (NTx 1 month), 13 (L-NAME 1 month), 12 (NTx 4 months), 10 (L-NAME 4 months).

To determine whether transient hypertension resulted in long-term cognitive deficits reported in aged individuals who experienced mid-life hypertension (Skoog et al., 1996; Tzourio et al., 2014), we used the Barnes maze task to assess the effects on learning, spatial memory, executive function, and cognitive flexibility one and 4 months after restoration of normotension. During the probe trials, latency to escape and search strategy complexity in the Barnes maze task demonstrate spatial memory (Illouz et al., 2016). One month after normotensive recovery, L-NAME-treated rats showed increased latency to escape (*p* = 0.006) and decreased search strategy complexity (*p* = 0.003) in the probe trials suggesting spatial memory impairment in comparison to NTx rats (Figures 2A,B). Four months after normotensive recovery, the latency to escape (*p* = 0.971) and search strategy complexity (*p* = 0.631) were not significantly different between NTx and L-NAME-treated rats (Figures 2A,B) suggesting recovery of spatial memory. Rats were assessed in the reversal Barnes maze task to examine executive function (Illouz et al., 2016). In the reversal trials, latency to escape was not significantly different between NTx and L-NAME-treated rats after 1 month (*p* = 0.631, ANOVA treatment effect) and 4 months (*p* = 0.692, ANOVA treatment effect) of normotensive recovery (Figure 2C) suggesting that executive function was not affected by the transient hypertensive insult. In contrast, search strategy complexity in the reversal trials was significantly lower in the L-NAME rats (*p* = 0.008, ANOVA treatment effect) at 1 month but not after 4 months of recovery (*p* = 0.132, ANOVA treatment effect) (Figure 2D). These results showed that cognitive flexibility similar to spatial memory was initially impaired by transient hypertension but recovered at least partially after an extended period of normotension. A closer examination of the different search strategies employed by these rats showed that recovery of cognitive flexibility was only partial: Although L-NAME-treated rats at 4 month employed increased search complexity compared to L-NAME rats at 1 month recovery (Figure 2E), they employed weak, less complex search strategies than NTx rats (Supplementary Figure 3) suggesting that deficits to cognitive flexibility remain. We confirmed this deficit by measuring the amount of time rats spent in the old escape quadrant during the last day of the reversal trials, another indicator of cognitive flexibility that requires the rats to replace the spatial memory encoded in the spatial trials with the new escape location. At 1 month recovery, L-NAME rats spent about twice the amount of time in the old escape quadrant (*p* = 0.004) compared to NTx rats, and this deficit persisted after 4 months recovery (*p* = 0.018) in comparison to NTx rats (Figure 2F). Taken together, the transient hypertensive event induced cognitive impairment in spatial memory and cognitive flexibility detected 1 month after return to normotension; deficits in spatial memory recovered back to baseline after prolonged recovery while deficits in cognitive flexibility remained.

### Vascular Compromise and Neuronal Loss After Transient Hypertension

**FIGURE 3:**
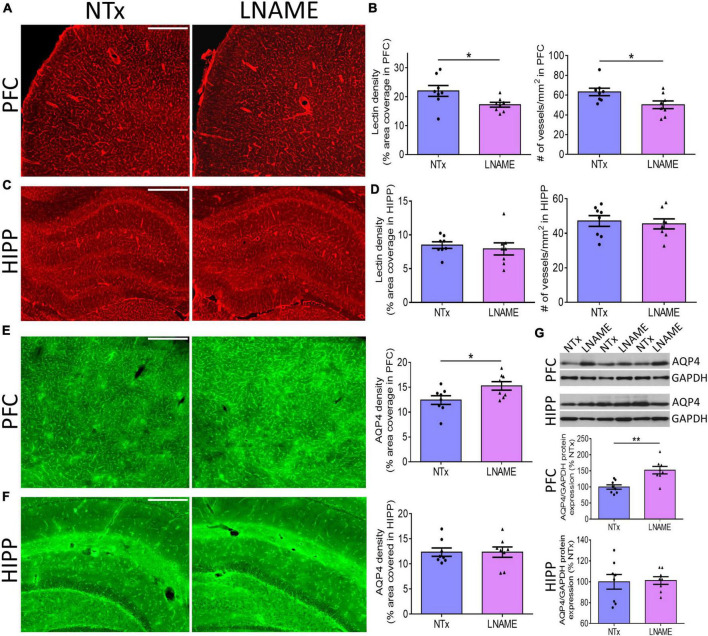
Transient hypertension results in irreversible compromise to the cerebrovasculature despite extended recovery. **(A)** Representative images of lectin fluorescence coverage in PFC; scale bar = 500 μm. **(B)** Quantification of lectin area coverage (left) and number of vessels (right) in PFC: Both lectin coverage (*p* = 0.036) and number of lectin-positive vessels (*p* = 0.032) are significantly lower in L-NAME group. **(C)** Representative images of lectin fluorescence coverage in HIPP; scale bar = 750 μm. **(D)** Quantification of lectin coverage (left) and number of vessels (right) in HIPP: Both lectin coverage (*p* = 0.593) and number of vessels (0.700) are not significantly different between groups (*p* = 0.593). **(E)** Representative images (left and middle) and quantification (right) of AQP4 fluorescence coverage in PFC: AQP4 immunoreactivity coverage is higher in L-NAME group (*p* = 0.036); scale bar = 500 μm. **(F)** Representative images (left and middle) and quantification (right) of AQP4 fluorescence coverage in HIPP: AQP4 immunoreactivity coverage is unchanged (*p* = 0.999). **(G)** Representative immunoblots (top) and immunoblot quantification of AQP4 protein expression normalized to GAPDH in PFC (middle) and HIPP (bottom); L-NAME increased AQP4 protein expression in PFC (*p* = 0.002) but not in HIPP (*p* = 0.879). Scale bar = 500 μm. Significance was calculated by *t*-test. * and ** denote *p* < 0.05 and *p* < 0.01 respectively; *n* = 8 rats per group.

To investigate the pathological underpinning of deficits in cognitive flexibility after transient hypertension, we hypothesized that both the cerebrovasculature and the brain parenchyma will have sustained irreversible damage. Blood vessels are critical for normal CNS function as the brain does not have an endogenous supply of oxygen or glucose. To probe the vasculature, we measured vessel density and structural integrity at the end of the recovery period, 9 months of age. We measured density of blood vessels by quantifying both fluorescence coverage of lectin staining and the total number of lectin-positive vessels: In prefrontal cortex (PFC) (*p* = 0.036), but not in hippocampus (HIPP) (*p* = 0.593), lectin coverage was 22 ± 9% lower in the L-NAME-treated rats compared to NTx rats (Figures 3A–D). Similarly, L-NAME decreased number of lectin-positive vessels by 21 ± 9% (*p* = 0.032) in PFC but not in HIPP (*p* = 0.700) (Figures 3B,D). To determine whether the structural integrity of cerebral vessels was affected, we probed for aquaporin-4 (AQP4), a water channel protein critical for maintenance of fluid exchange at the blood brain barrier and altered in chronically hypertensive rats (Tomassoni et al., 2010). We found a 23 ± 10% increase in PFC AQP4 fluorescence coverage in L-NAME-treated rats compared to NTx rats (*p* = 0.036) suggesting compromised communication between blood and brain parenchyma (Figure 3E). Similar to vessel density, AQP4 fluorescence coverage in HIPP was unchanged in L-NAME-treated rats compared to NTx rats (*p* = 0.999) (Figure 3F). Immunoblotting of AQP4 protein expression (Figure 3G) showed similar results such that L-NAME upregulated AQP4 protein expression in PFC (*p* = 0.002) but not in HIPP (*p* = 0.879).

**FIGURE 4:**
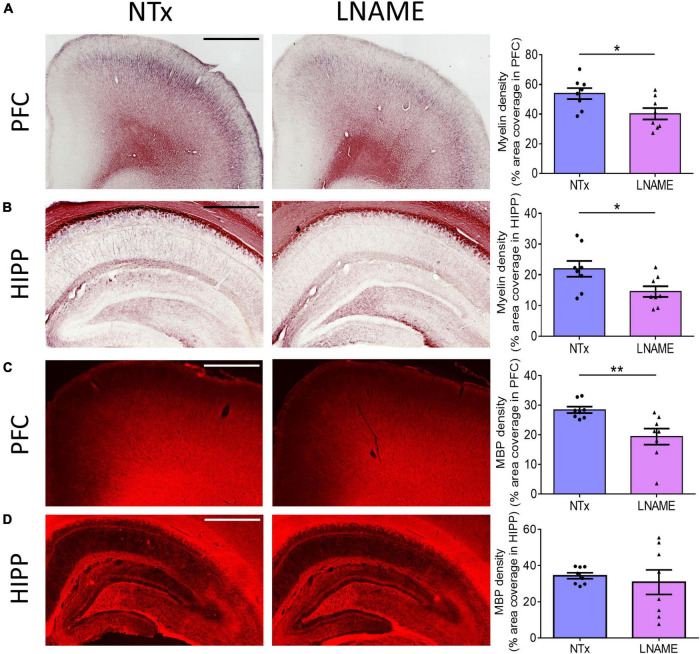
Transient hypertension promotes irreversible loss of myelin density. **(A)** Representative images (left and middle) and quantification (right) of myelin density in PFC: L-NAME-treated rats had lower myelin density (*p* = 0.023); scale bar = 1,250 μm. **(B)** Representative images (left and middle) and quantification (right) of myelin density in HIPP: L-NAME-treated rats had lower myelin density (*p* = 0.031); scale bar = 750 μm. **(C)** Representative images (left and middle) and quantification (right) of MBP fluorescence coverage in PFC: L-NAME treatment decreased coverage of MBP immunoreactivity (*p* = 0.008); scale bar = 1,000 μm. **(D)** Representative images (left and middle) and quantification (right) of MBP fluorescence coverage in HIPP: MBP immunoreactivity coverage was unchanged (*p* = 0.621); scale bar = 875 μm.

**FIGURE 5:**
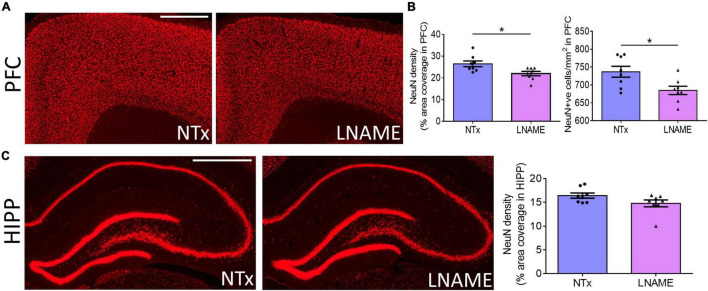
Transient hypertension promotes irreversible loss of neuronal density. **(A)** Representative images of NeuN fluorescence coverage in PFC; scale bar = 750 μm. **(B)** Quantification of NeuN fluorescence coverage (left) and number of NeuN-positive cells (right) in PFC: L-NAME treatment decreased both coverage of NeuN immunoreactivity (*p* = 0.017) and number of NeuN-positive cells (*p* = 0.017). **(C)** Representative images (left and middle) and quantification (right) of NeuN fluorescence coverage in HIPP: L-NAME treatment induced a small but statistically insignificant decrease of NeuN coverage (*p* = 0.093); scale bar = 1,000 μm. * denotes *p* < 0.05; *n* = 8 rats per group.

Compromised vessel density and structure implicates downstream damage to the brain parenchyma. Unresolved hypertension is a known risk factor for development of white matter injury (Liao et al., 1996; Basile et al., 2006). Here, myelin-specific Black Gold II staining showed that in both PFC (*p* = 0.023) and HIPP (*p* = 0.031), transient hypertension significantly decreased myelin density by 25 ± 10% and 34 ± 14%, respectively (Figures 4A,B). Corroboratively, hypertension also decreased density of myelin basic protein (MBP) immunoreactivity by 32 ± 10% in PFC (*p* = 0.008), but not in HIPP (*p* = 0.621) (Figures 4C,D). In addition to myelin damage, we probed for neuronal loss. Using the neuron-specific marker NeuN, we found that compared to NTx rats, L-NAME-treated rats had 17 ± 6% lower density of NeuN immunoreactivity (*p* = 0.017) (Figure 5A) and less NeuN-positive cells (*p* = 0.017) (Figure 5B) in PFC. Neuronal loss in HIPP in contrast was not as severe (10 ± 6% lower) and not statistically significant (*p* = 0.093) (Figure 5C). Taken together, rats that sustained 1 month of hypertension suffered irreversible damage to both cerebral vessels and neurons in the PFC after 4 months of normotensive recovery.

### Altered Network Function and Prefrontal Cortical-Hippocampal Interactions

**FIGURE 6:**
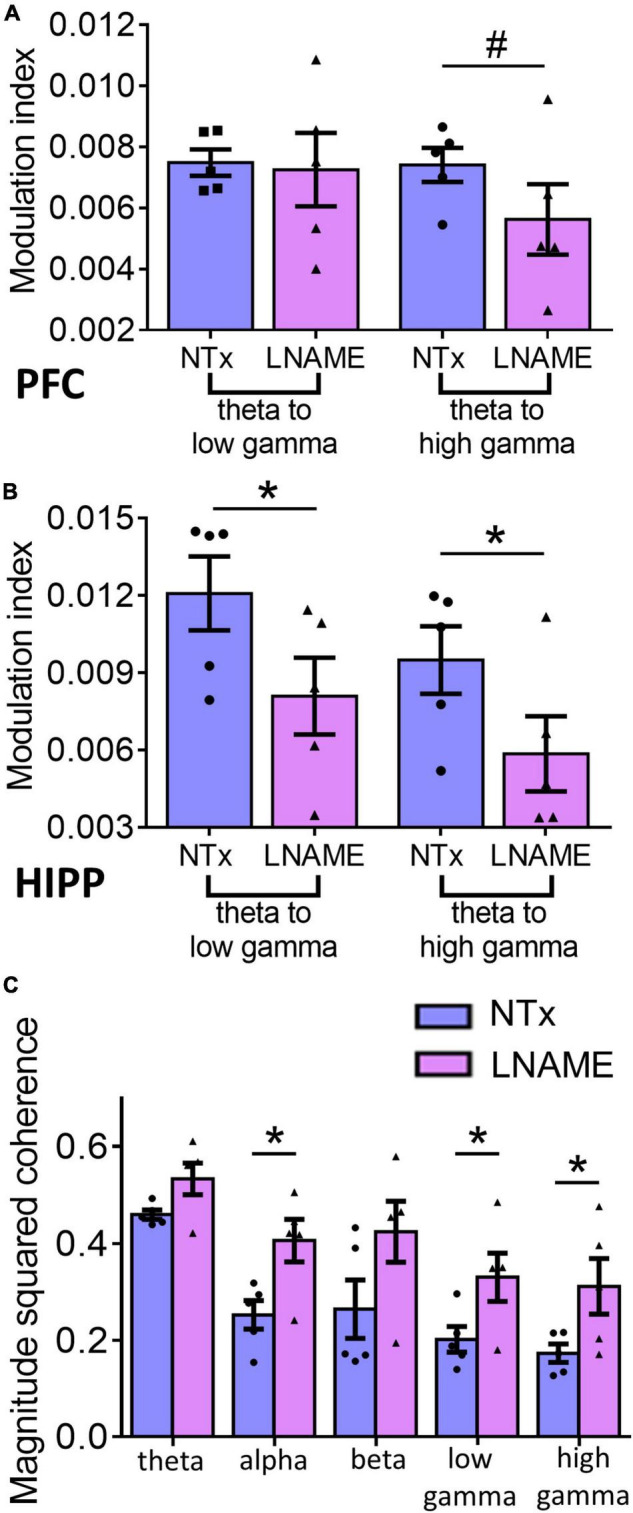
Electrophysiological recordings show that injuries sustained from transient hypertension translate to impaired neuronal network function. **(A)** In PFC, theta to low gamma PAC was not significantly different between NTx and L-NAME rats (*p* = 0.78), while theta to high gamma PAC was significantly smaller in L-NAME-treated rats (*p* = 0.056). **(B)** In HIPP, both theta to low gamma PAC (*p* = 0.016) and theta to high gamma PAC (*p* = 0.021) were smaller in L-NAME treated rats. **(C)** MSC between PFC and HIPP: In alpha (*p* = 0.018), low gamma (*p* = 0.042), and high gamma bands (0.043), MSC was significantly increased in L-NAME-treated rats compared to NTx rats. In contrast, in theta (*p* = 0.17) and beta (*p* = 0.19) bands, MSC was not significantly different between L-NAME and NTx rats. * denotes *p* < 0.05; # denotes *p* < 0.06; *n* = 5 rats per group.

Since vascular and neuronal injuries were observed in PFC and HIPP, we used electrophysiological measures to detect neuronal network function and complement the resting state measurements with neuronal excitability and the integrity of hippocampal-prefrontal connectivity. To assess within region network function, we assessed phase amplitude coupling as a measure of neuronal integration. Interestingly, while pathologically PFC sustained more hypertension-induced damage, the HIPP exhibited greater network deficits. In the PFC (Figure 6A), theta to high gamma phase amplitude coupling was significantly smaller, 24.4 ± 2.8%, in the L-NAME-treated rats (0.00562 ± 0.00012) when compared to NTx rats (0.00741 ± 0.00015; *p* = 0.056), while theta to low gamma phase amplitude coupling was not significantly affected (0.00748 ± 0.00014 in NTx vs. 0.0072 ± 0.00014 in L-NAME, *p* = 0.78). Contrastingly, in the HIPP (Figure 6B), we observed that theta to low gamma phase amplitude coupling was 33.1 ± 3.5% smaller in L-NAME-treated rats (0.0125 ± 0.00025) compared to NTx rats (0.0086 ± 0.00016; *p* = 0.016), while theta to high gamma phase amplitude coupling was 38.3 ± 5.7% lower in the L-NAME-treated group (0.0094 ± 0.00017) when compared to the NTx group (0.0058 ± 0.00012, *p* = 0.021). To test the connectivity between PFC and HIPP, we assessed magnitude squared coherence between the oscillations at bands of interest between the two areas. We found that magnitude squared coherence was significantly increased in hypertensive rats when compared to NTx rats in alpha (71.5 ± 3.4%, 0.21 ± 0.016 in NTx vs. 0.36 ± 0.011 in L-NAME, *p* = 0.018), low gamma (57.8 ± 7.3%, 0.19 ± 0.0026 in NTx vs. 0.31 ± 0.004 in L-NAME, *p* = 0.042), and high gamma (70.1 ± 9.1%, 0.17 ± 0.023 in NTx vs. 0.29 ± 0.042 in L-NAME, *p* = 0.043) (Figure 6C). We observed no statistical difference in magnitude squared coherence between the two groups in the theta (0.43 ± 0.019 in NTx vs. 0.49 ± 0.018 in L-NAME, *p* = 0.17) and beta (0.25 ± 0.007 vs. 0.38 ± 0.008 in L-NAME, *p* = 0.19) bands (Figure 6C). Collectively, these results show that vascular and neuronal injuries sustained from transient hypertension are accompanied by neuronal network dysfunction in both PFC and HIPP. More importantly, increased coherence in several frequency bands between the two regions demonstrate that network interactions between PFC and HIPP have been irreversibly altered despite an extended period of normotensive recovery.

## Discussion

The current study modeled transient hypertension to reflect the extended period of normotensive recovery present in most hypertensive patients. Our previous work examining the effects of transient hypertension had only 1 month of normotensive recovery (Lai et al., 2021). Here, we extended the normotensive recovery time to 4 months to more closely resemble clinical hypertension. A notable limitation of our study is that at 4 months of age when hypertension was induced, rats have been proposed to represent an age similar to young adults rather than those at mid-life. Nevertheless, 4 months of recovery has been proposed to equate to ∼12 human years (Sengupta, 2013) representing a time window in which to differentiate the neuronal circuits most vulnerable to hypertensive insult over a long recovery period. Another limitation worth noting is that we measured blood pressure under isoflurane anesthesia which is known to depress blood pressure in a dose dependent manner (Yang et al., 2014). Since taking blood pressure measurements in awake rats would have necessitated restraint and thereby elicited varying level of stress and varying levels of blood pressure elevation (Zimmerman and Frohlich, 1990), and since we are able to regulate the concentration of isoflurane, we opted to measure under anesthesia.

We showed that interaction between PFC and HIPP was irreversibly affected by transient hypertension despite 4 months of recovery. Some aspects of the PFC-HIPP network did, however, recover in a region-specific manner. Specifically, both the cerebrovasculature and brain parenchyma showed a degree of resiliency to chronic hypertensive insult and partially recovered after 4 months. With respect to vessel density, myelin integrity, and neuronal survival, we showed that damage was primarily in PFC but not in HIPP. On the other hand, electrophysiology showed the opposite trend such that theta to gamma phase amplitude coupling in HIPP failed to recover at 4 months whereas theta to gamma phase amplitude coupling in PFC did. A possible explanation for these results is that loss of vessels and neurons in PFC may have induced circuit remodeling both within PFC and in other brain regions connected to PFC, particularly HIPP, which manifested in recovery of theta to gamma phase amplitude coupling in PFC despite loss of blood vessels and neurons. By the same token, remodeling could also produce detrimental results where theta to gamma phase amplitude coupling in HIPP becomes impaired despite normal vascular and neuronal densities. This form of aberrant remodeling is best illustrated by our results measuring magnitude squared coherence between PFC and HIPP. We observed increased coherence between the two regions in three different frequency bands. While increased coherence is most often observed when the animal successfully consolidates or learns a memory task (Sigurdsson and Duvarci, 2016), a baseline coherence that is elevated for an extended period of time is not beneficial by default. Increased baseline coherence has been associated with various pathological conditions including seizures and schizophrenia (Andreou et al., 2015; Kitchigina, 2018), so it is plausible that the same phenomenon underlies reduced cognitive performances we observed in this study.

Results from the Barnes maze readouts support the notion that transient hypertension induced circuit remodeling with both compensatory and detrimental effects. PFC-HIPP pathways and interactions are critical for the normal functioning of spatial memory, executive function, and cognitive flexibility (Jin and Maren, 2015). Executive function was spared from hypertensive insults but spatial memory and cognitive flexibility were both impaired initially. Deficits in spatial memory recovered after 4 months of normotension despite a significant loss of blood vessels and neurons in PFC. Similarly, deficits in cognitive flexibility also recovered but only partially, suggesting that circuit remodeling in either PFC or HIPP was insufficient. The precise cellular mechanisms underlying this remodeling require further investigation.

With respect to dementia, vascular risk factors including hypertension remain an area of high importance due to their modifiable and tractable nature. Effective management of hypertension risk may lessen the societal burden on the imminent dementia epidemic. Time and strength of hypertension management are crucial: There is increasing evidence that managing blood pressure earlier in life leads to significantly improved later outcomes regarding brain damage and dementia risk (Qiu et al., 2005; Rouch et al., 2015). With respect to treatment strength, the SPRINT-MIND trials (SPRINT MIND Investigators for the SPRINT Research Group, Nasrallah et al., 2019a; SPRINT MIND Investigators for the SPRINT Research Group, Williamson et al., 2019b) concluded that compared to standard hypertensive management, intensive lowering of blood pressure was associated with reduced risk of mild cognitive impairment and less white matter lesions. Notably, these studies did not discern the differences between specific hypertensive medications nor did they account for subjects with other dementia risk factors such as stroke, diabetes and other vascular diseases. Further investigation is thus needed to determine the most effective treatment paradigm. Our results here have provided key mechanistic links between hypertensive events and increased dementia risk and should aid the efforts to optimize improved treatment strategies.

## Data Availability Statement

The raw data supporting the conclusions of this article will be made available by the authors, without undue reservation.

## Ethics Statement

The animal study was reviewed and approved by the Animal Care Committee of the Sunnybrook Health Sciences Center.

## Author Contributions

AL, JM, and BS designed the study. PB conducted and analyzed the electrophysiological experiments. CM and MH conducted and analyzed the behavior experiments. AL performed the tissue-processing experiments and drafted the manuscript. JM and BS edited the manuscript. All authors contributed to the article and approved the submitted version.

## Conflict of Interest

The authors declare that the research was conducted in the absence of any commercial or financial relationships that could be construed as a potential conflict of interest.

## Publisher’s Note

All claims expressed in this article are solely those of the authors and do not necessarily represent those of their affiliated organizations, or those of the publisher, the editors and the reviewers. Any product that may be evaluated in this article, or claim that may be made by its manufacturer, is not guaranteed or endorsed by the publisher.

## Footnotes

1
https://www.cs.bris.ac.uk/Research/MachineLearning/pac/

2
http://www.R-project.org
