# Supplementary material for: Compromised Cortical-Hippocampal Network Function From Transient Hypertension: Linking Mid-Life Hypertension to Late Life Dementia Risk
Source: Front Neurosci. 2022 Jun 23;16:897206. doi: 10.3389/fnins.2022.897206 (PMC9260147; doi:10.3389/fnins.2022.897206)
Supplement: Supplementary file 1 [file Data_Sheet_1.PDF]

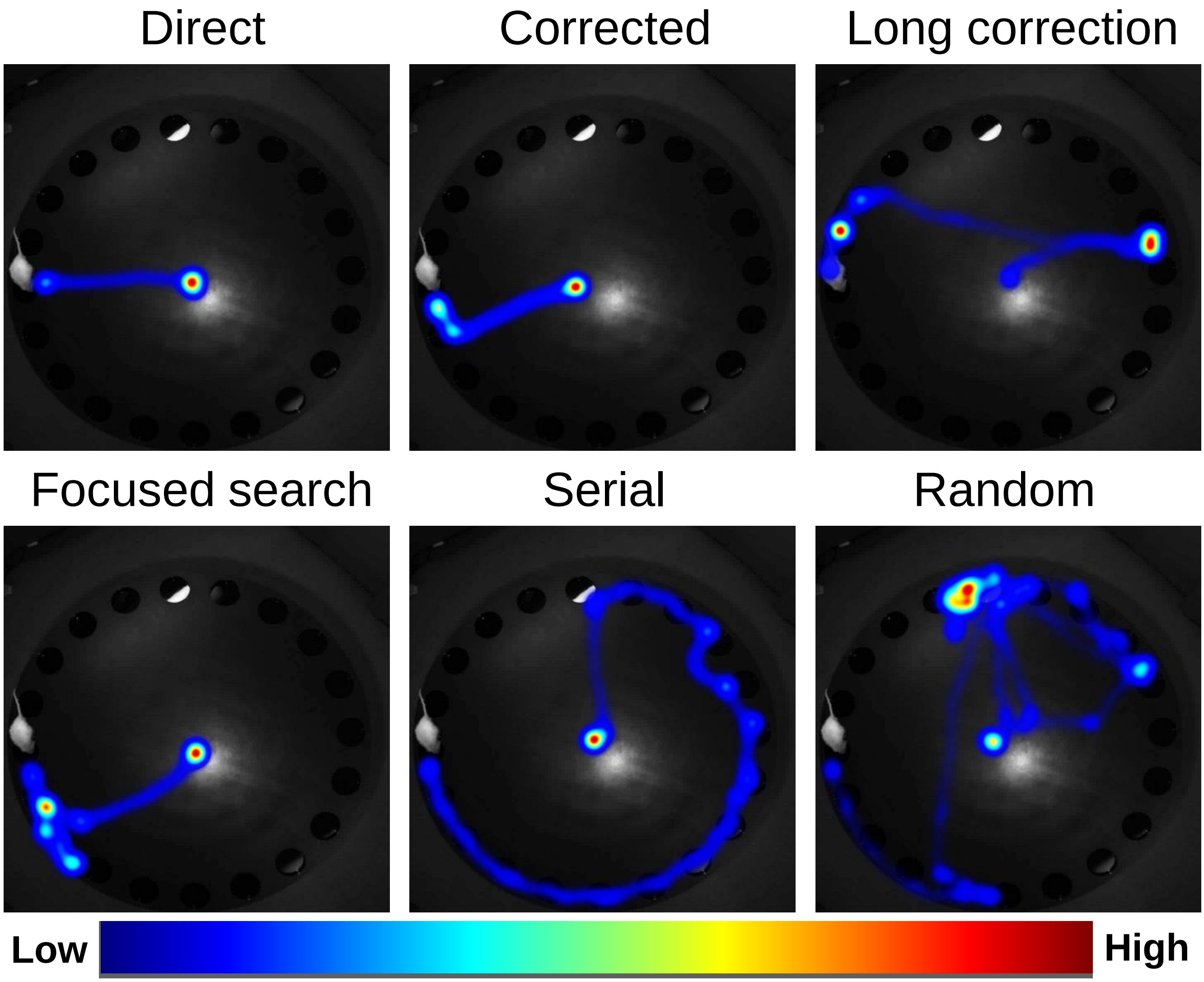

Figure S1. Representative heatmaps of the six search strategies taken from the learning trials of all of the experimental groups.

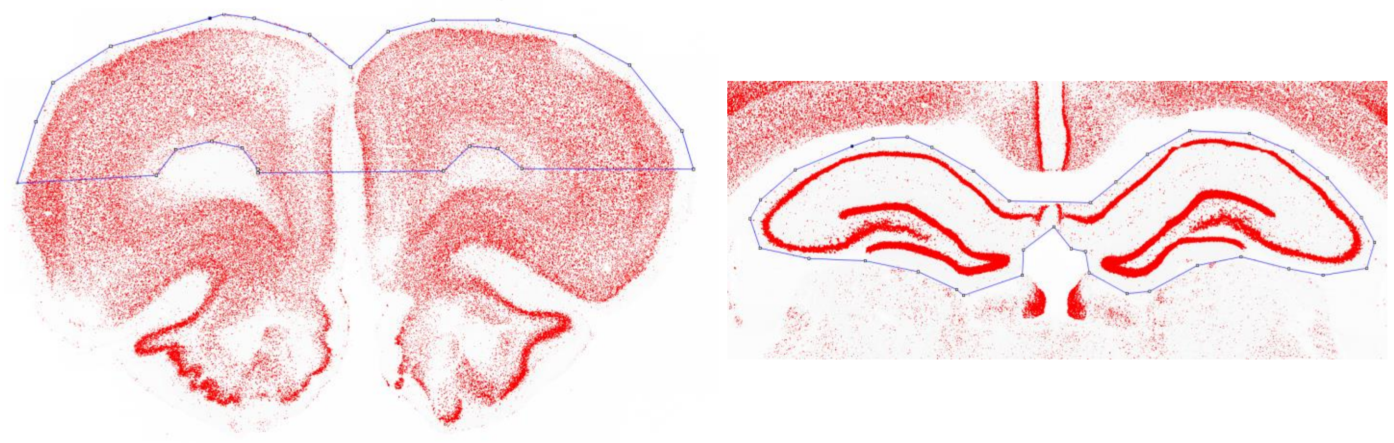

Figure S2. Representative region of interest drawn (blue outline) to quantify a thresholded image containing the prefrontal cortex (left) and hippocampus (right).

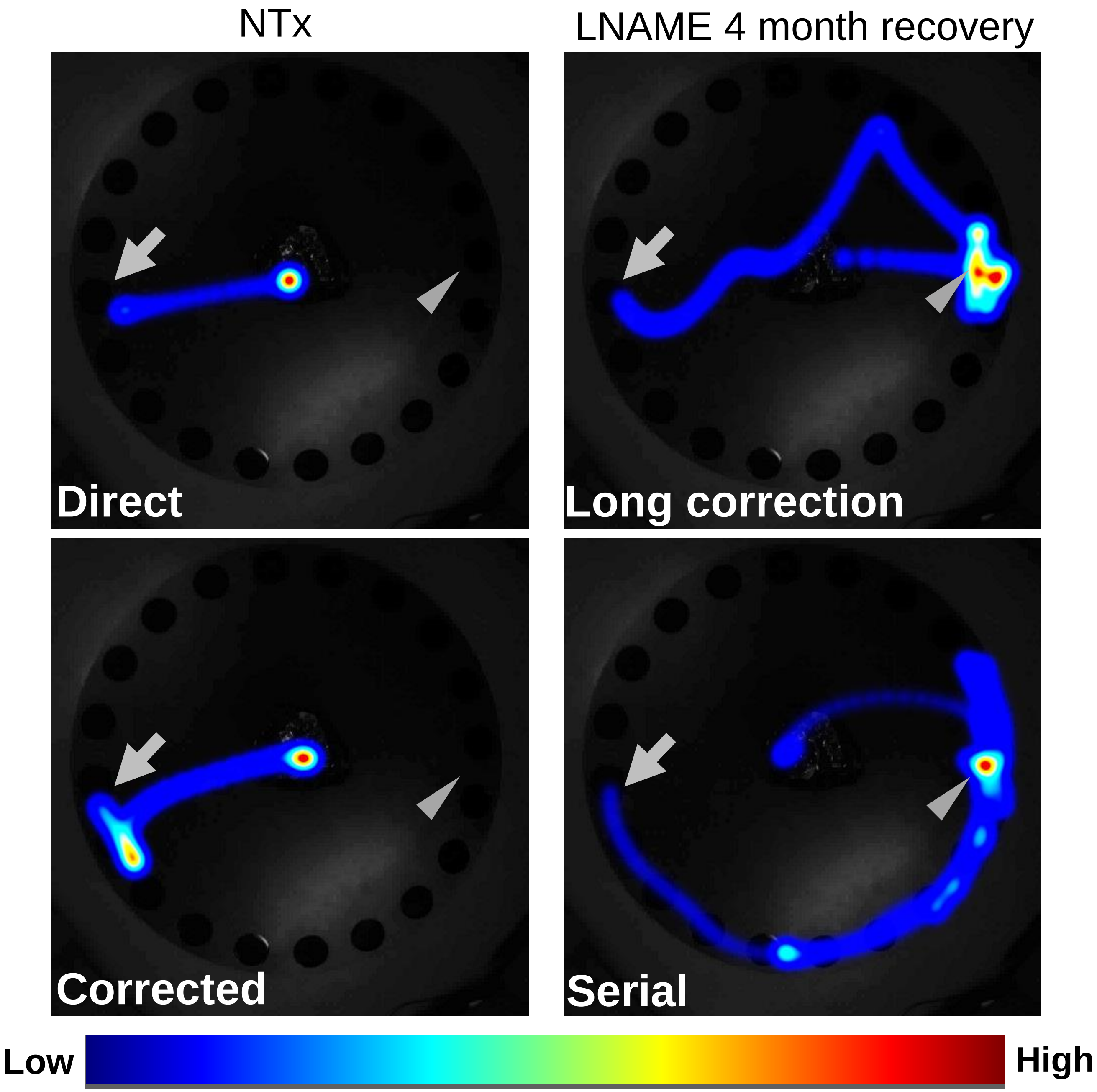

Figure S3. Representative heatmaps of the two search strategies most used by NTx (left) and LNAME rats (right) during the reversal trials. Arrowheads indicate the old escape holes while arrows mark placement of the new holes.
